# Chordae tendineae rupture during a helix-fixation leadless pacemaker implantation

**DOI:** 10.1093/ehjcr/ytag090

**Published:** 2026-02-03

**Authors:** Tomoki Fukui, Miwa Miyoshi, Nobuyuki Ogasawara

**Affiliations:** Department of Cardiology, Japan Community Healthcare Organization Osaka Hospital (Sub-hospital Name: Osaka Byoin), 4-2-78, Fukushima, Fukushima-Ku, Osaka 553-0003, Japan; Department of Cardiology, Japan Community Healthcare Organization Osaka Hospital (Sub-hospital Name: Osaka Byoin), 4-2-78, Fukushima, Fukushima-Ku, Osaka 553-0003, Japan; Department of Cardiology, Japan Community Healthcare Organization Osaka Hospital (Sub-hospital Name: Osaka Byoin), 4-2-78, Fukushima, Fukushima-Ku, Osaka 553-0003, Japan

## Case description

**Figure 1:**
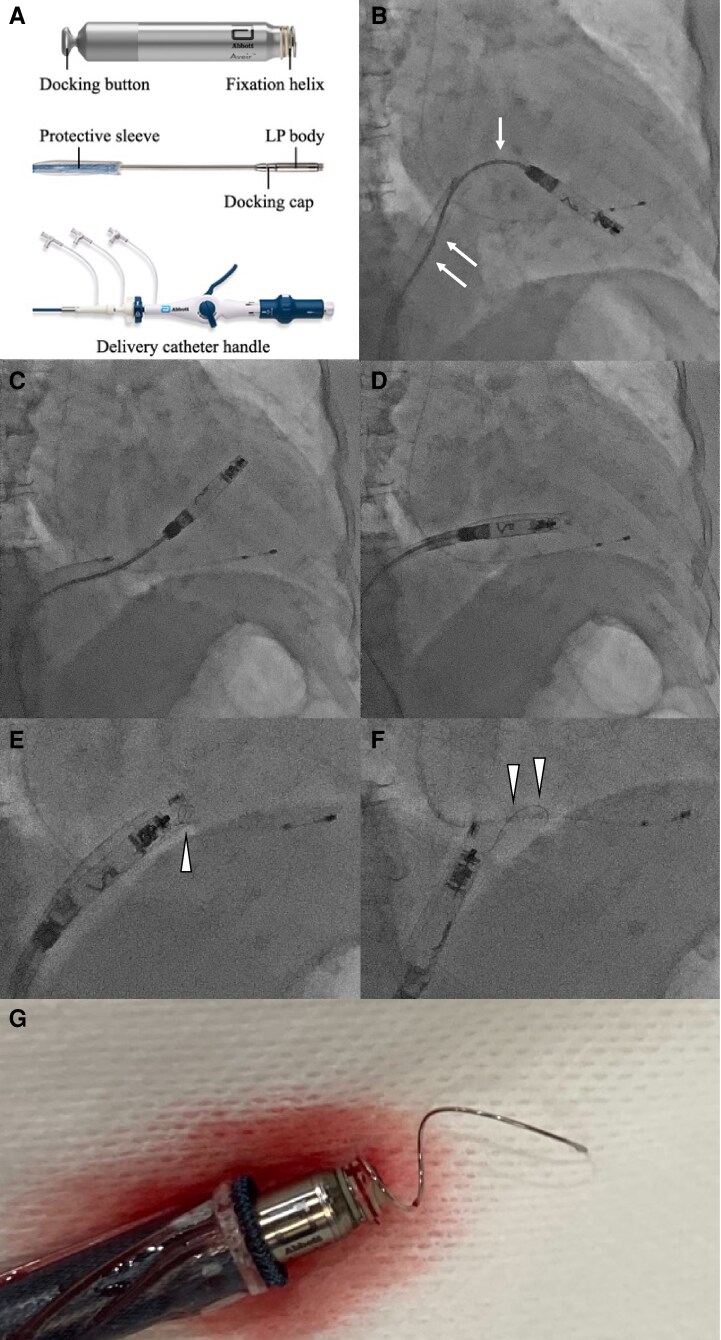
(*A*) The Aveir™ VR single-chamber leadless pacemaker and leadless pacemaker delivery system. (*B*) Sleeve-out refers to the process in which the protective sleeve covering the leadless pacemaker body is retracted, thereby exposing the leadless pacemaker body. The leadless pacemaker delivery catheter showed bending that was likely caused by accumulated catheter torque and strong backup force (arrows). (*C*) The leadless pacemaker was dislocated from the right ventricular apical septum to the right ventricular outflow tract. (*D*) The leadless pacemaker became fully covered with a protective sleeve. (*E*) Pulling of the leadless pacemaker and delivery catheter resulted in an elongation of the helix. Helix elongation was first observed around the right atrium and tricuspid valve (arrow). (*F*) Complete helix elongation was noted around the right atrium and inferior vena cava (arrows). (*G*) Helix elongation was confirmed.

The Aveir™ VR single-chamber (*Figure 1A*) and DR dual-chamber leadless pacemakers (LPs) (Abbott, Chicago, IL) were approved in 2022 and 2025, respectively. With advantages such as a high procedural success rate, retrievability, and long battery life, this novel helix-fixation system has gained widespread adoption.^1–4^ Consequently, the incidence of device-related complications is expected to increase, including cardiac perforation, dislodgement, stretched helix, and tricuspid injury.^3–6^ To our knowledge, this is the first report describing simultaneous chordae tendineae rupture and helix stretching during implantation.

An 89-year-old woman was referred for evaluation of syncope caused by an atrioventricular block lasting 8 s. Her medical history included paroxysmal atrial fibrillation and chronic kidney disease. A temporary pacing lead was emergently inserted. Considering her advanced age and frailty, LP implantation was selected for its lower complication risk.^3^ Subsequently, helix-fixation LP implantation via the femoral vein was performed. The LP was initially positioned at the apical septum and was screwed in and out twice because of unacceptable thresholds and impedance values. After a slight positional adjustment and subsequent sleeve-out, the LP drifted from the right ventricular apical septum to the right ventricular outflow tract (*Figure 1B* and *C*). The LP became fully covered with a protective sleeve; however, marked resistance was encountered during advancement and withdrawal of both the LP body and the delivery catheter (*Figure 1D*). Pulling of the LP and delivery catheter resulted in an elongation of the helix (*Figure 1E* and *F*; Supplementary material online, *Video S1*). After removal of the LP and delivery catheter from the body, helix elongation and ruptured chordae tendineae were visually confirmed (*Figure 1G*). A new LP was safely implanted using a different delivery catheter. Transthoracic echocardiography revealed progression of tricuspid regurgitation from moderate before the procedure to severe after the procedure. No cardiac events occurred during the 4-month follow-up. Tricuspid regurgitation improved to a moderate level over time.

A previous report described tricuspid valve entrapment by the delivery catheter, with tissue caught between the docking button and cap.^5^ In our case, the tissue was entrapped by the helix itself, leading to helix elongation and chordae tendineae rupture. The US Food and Drug Administration database reported 11 events involving stretched helices between 2022 and 2023,^6^ highlighting the clinical relevance of this complication. Movement of an exposed LP poses a risk of tricuspid valve entanglement, as seen in our case. Repositioning inherently involves multiple LP exposures during the sleeve-out process, and 16.8% of cases reportedly required at least one device repositioning during implantation.^3^ Leadless pacemaker translocation in our case was likely caused by accumulated catheter torque and strong backup force, compounded by repeated rotation and advancement of the delivery catheter, limited right ventricular space, and catheter stiffness. Avoiding excessive force during delivery catheter manipulation and minimizing sleeve-out exposure are essential to reduce such complications. This case emphasizes the importance of careful procedural planning and awareness of potential helix-related complications during helix-fixation LP implantation.

## Supplementary Material

ytag090_Supplementary_Data

## Data Availability

The data underlying this article will be shared on reasonable request to the corresponding author.
